# The Role of 3D Printed Spine Model in Complex Spinal Deformity Surgery; an Experience With a Case, Technical Notes and Review of the Literature

**DOI:** 10.1002/ccr3.70675

**Published:** 2025-08-03

**Authors:** Hamed Hanif, Negin Safari, Reza Sharifi, Nima Ostadrahimi, Alaaeldin Ahmad

**Affiliations:** ^1^ Neurosurgery Department Tehran University of Medical Sciences, Sina Hospital Tehran Iran; ^2^ Tehran University of Medical Sciences Tehran Iran; ^3^ Palestine Polytechnic University Hebron Palestine

**Keywords:** 3D printed spine model, complex spinal deformity, congenital scoliosis, split cord malformation, stereolithography

## Abstract

Complex spinal deformity in adults, often from neglected congenital deformities, poses surgical challenges. Understanding the 3D anatomy is crucial. A 3D‐printed spinal model aids pre‐operative planning, intra‐operative navigation, and education. It reduces surgery time, bleeding, and X‐ray dose, while improving screw placement accuracy and navigation of intra‐spinal pathologies.

## 1 Introduction

Complex spinal deformities in adults can develop as long‐term consequences of congenital spinal pathologies that were neglected during childhood and adolescence [1, 2, 3]. These patients typically present to the clinic with concerns related to body shape and aesthetics, the progression of degenerative spinal disease, or neurological symptoms [4]. Corrective spinal surgery in these patients is particularly challenging in older ages due to the severity of the deformity, spinal rigidity, and decreased plasticity of neural tissues for repositioning. Accompanying pathologies such as split cord malformation (SCM) and other types of tethered cord syndrome present major challenges that must be addressed before any corrective spinal maneuvers. Precise localization of intraspinal pathologies in the presence of unusual spinal anatomy can be difficult. Various tools for spinal navigation may assist a surgeon in enhancing the safety and efficacy of complex spinal deformity surgery.

A significant preoperative challenge in complex spinal deformity is achieving a three‐dimensional understanding of the deformity based on two‐dimensional imaging [5]. The use of 3D models enhances the spatial understanding of spinal dimensions, distances, and coordinates [6, 7, 8]. Despite advancements in medical imaging resolution, image processing and 3D reconstruction, and the development of neuronavigation and augmented reality technologies, physical 3D models of the spine on a 1:1 scale continue to hold incomparable clinical and educational value [9].

Our study demonstrates the benefits of 3D printing through a case of complex spinal deformity that underwent surgical correction using this method. We present a literature review and provide detailed technical notes on the printing process and surgical techniques utilized.

## 2 Case Presentation

A 38‐year‐old woman presented to our clinic with gait imbalance and asymmetrical appearance of the trunk and limbs. Examination revealed severe asymmetry of the waist, pelvis, and shoulders, with left‐side costoiliac impingement causing a deep waist crease and skin hyperpigmentation. Neurological examinations showed no significant findings.

## 3 Methods: Investigations and Treatment

**FIGURE 1:**
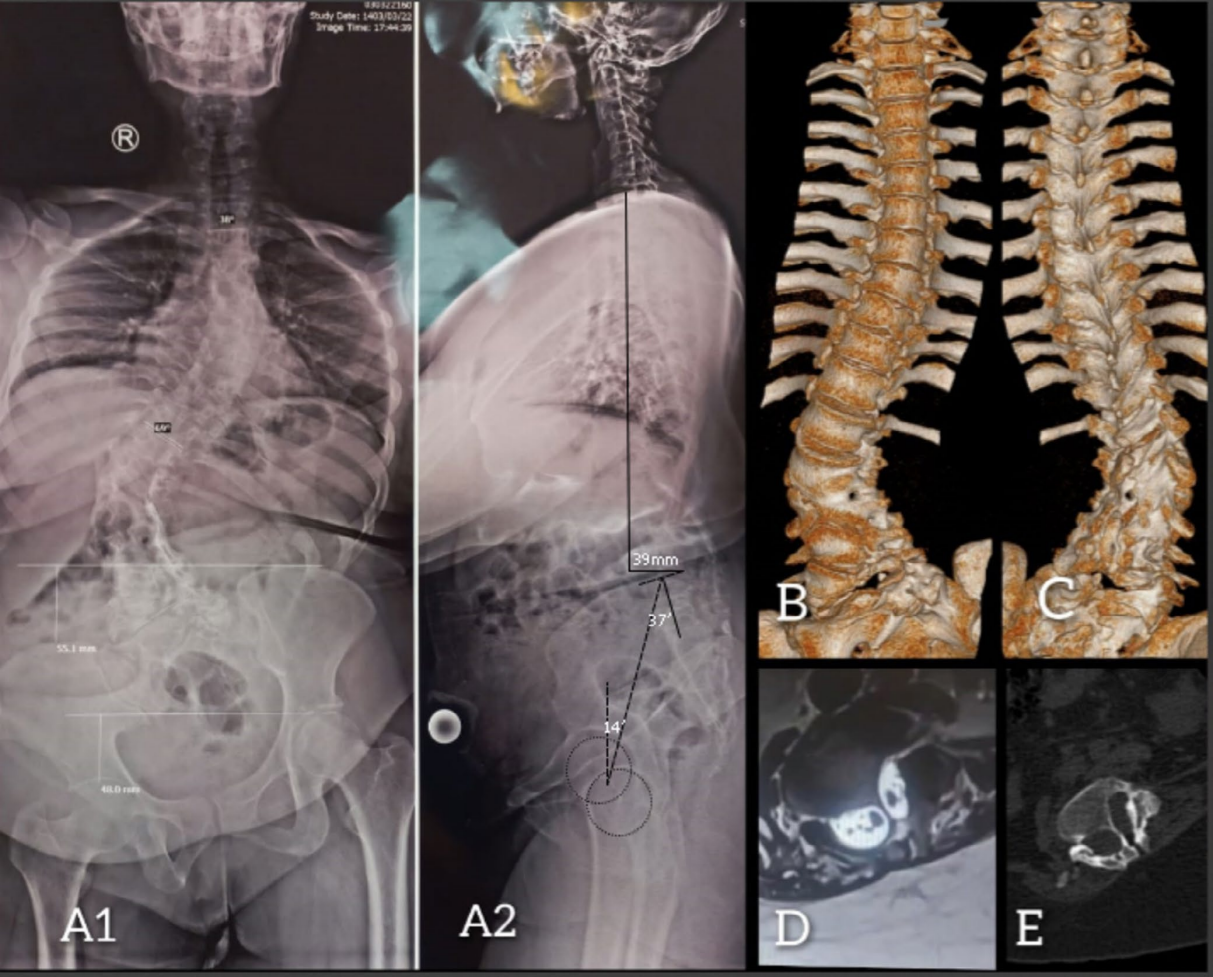
(A) 36 in. scoliosis radiography shows 70° scoliosis in thoracolumbar region. A1 coronal view: Shoulders height difference is 26 mm, pelvic obliquity about 55 mm, and femoral head height difference is 48 mm. Left side waist crease is prominent. A2 Sagittal view: Acceptable sagittal vertical alignment without compensatory pelvic retroversion. (B) and (C) show Anterior and posterior view of the 3D reconstructed CT scan of the spine. (D) and (E) shows type 1 split cord malformation (SCM).

**FIGURE 2:**
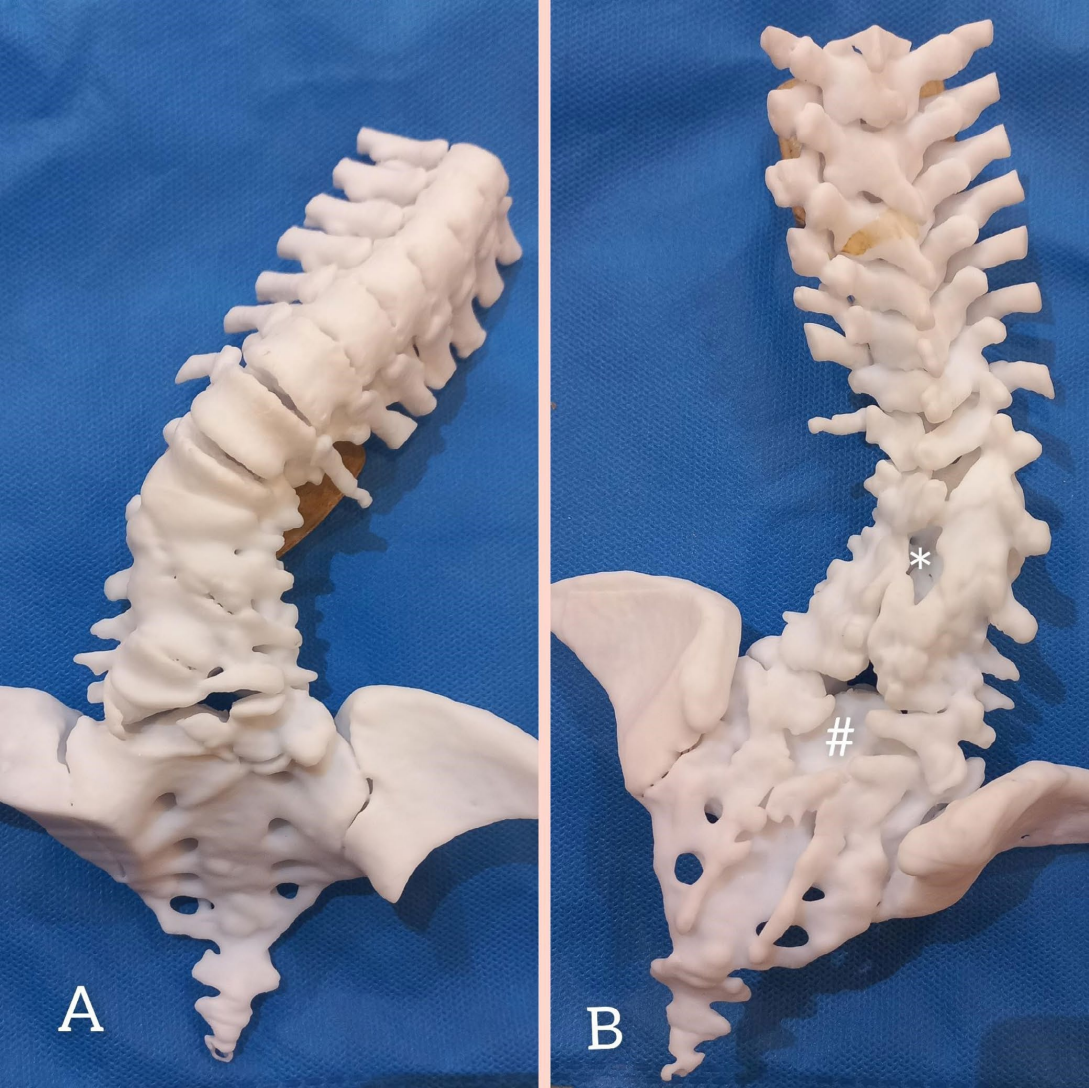
3D printed model of the patient's spine, from mid thoracic to coccyx. (A) Anterior view; shows complex vertebral anomalies including multiple defects of formation and segmentation, resulted in a dysmorphic rigid bony block instead of normal lumbar spine. Two hemi‐vertebras, one in the lumbosacral junction, and on in the thoracolumbar junction is identifiable in this model. (B) posterior view; shows defective posterior elements of the lumbar spine. (#) and (*) show two dysraphic areas of the lumbar region, that the bony SCM lies in between.

Preoperative Evaluations: Standard preoperative evaluations, including X‐ray, CT, MRI, and cardiopulmonary workups, were performed. Imaging revealed a complex coronal deformity on 2D radiography and 3D reconstruction CT scan (Figure 1) that could not be comprehensively understood as a 3D anatomic concept. Given the complexity of the deformity and the presence of multiple concurrent pathologies on CT and MRI (hemivertebra, dysraphism, diastematomyelia), we decided to print a physical model of the spine to better understand the dimensions and spatial proportions of the deformity (Figure 2). We placed the 3D model next to us in the operating room and used it to observe the necessary landmarks for the surgery. Especially, it was very helpful in easily locating the SCM site.

### 3.1 Preparation of the 3D Printed Model

Spine and pelvic segmentation were performed using the segment editor module of the 3D Slicer software [10]. The marching cubes algorithm was then used to generate the three‐dimensional complete model. An expert user determined the weak borders and osseous regions. The internal part of the model and border gaps were manually filled in the software to ensure consistent quality for 3D printing. The model was then clipped to the desired region for 3D printing. Using stereolithography (SLA) technology with 0.2 mm precision, the spine from T8 to the coccyx and the posterior parts of the iliac crests were included in the 3D printed model.

### 3.2 Surgical Procedure

- Step 1: After general anesthesia and neuromonitoring setup, a vertical midline incision was made in the lumbosacral area, and the paraspinal muscles were carefully shaved. The spine from T7 to the iliac wings was exposed. The landmarks observed in the surgical field matched the printed model. Considering the position of the SCM in the 3D model, we started the laminectomy from the caudal dysraphic area (Figure 2, #) and continued to the rostral dysraphic area (Figure 2, *). The SCM bony bar was easily located and resected.
- Step 2: Based on MRI findings, a midline durotomy was performed under the microscope in the lower lumbosacral area, and the spinal cord was detethered under the guidance of trigger‐EMG.
- Step 3: Pedicle screws were placed in the segments above and below the hemivertebrae of the thoracolumbar junction. The entry points and trajectories of the screws were selected based on the 3D printed model and placed under the guidance of C‐arm.
- Step 4: As we had planned based on the 3D model, hemivertebrectomy was performed, followed by discectomy on the concave side. The osteotomy site was closed using manual chest pushing and compression maneuvers. Decortication and fusion were performed. A subfascial drain was placed. The muscles, fascia, subcutaneous tissue, and skin were closed.

This one‐stage surgery lasted four and a half hours, with a total blood loss of 1200 cc. The patient was able to walk 24 h post‐operation and was discharged on the fourth day. Apart from moderate paresthesia in the right L3 dermatome, there were no major neurological deficits.

## 4 Follow Up and Outcome

**FIGURE 3:**
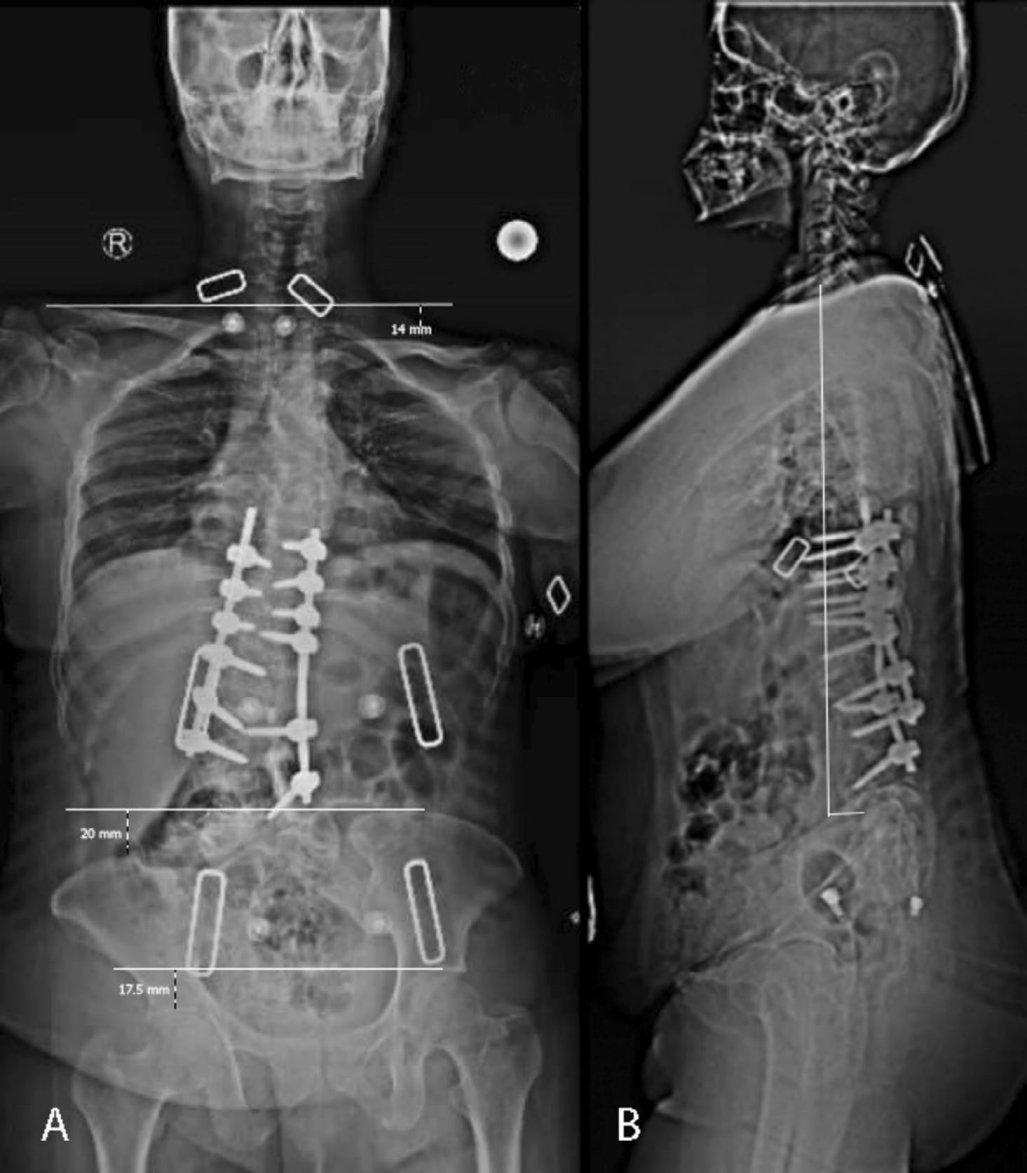
Post‐operative standing scoliosis film. (A) Antero‐posterior view shows improvement in shoulder balance from 26.1 mm to 14 mm; pelvic obliquity from 55 mm to 20 mm; and femoral head reference line from 48 mm to 17.5 mm. (B) Lateral view: No significant change was observed in sagittal parameters.

In a 36‐in. radiograph 1 month after surgery (Figure 3), there was a 60% overall improvement in shoulder balance, pelvic obliquity, and femoral head height difference. The patient was satisfied with the clinical outcome, reporting improvements in gait, trunk balance, and symmetric waist creases.

## 5 Discussion

Since the 1990s, 3D printing technology, also known as rapid prototyping, has been introduced to create physical models from anatomical images. Since then, it has been used in many orthopedic and spine surgeries [11, 12]. 3D printed spine models can be utilized in deformity surgeries, hemivertebra resection, cranio‐cervical junction surgeries, minimally invasive spine surgeries, and spinal oncology surgeries to produce custom‐made implants. In the following paragraphs, we will focus more on the advantages and applications of 3D spine models in spinal deformity surgeries. Table 1 shows the summary of studies that used various 3D printing techniques for the treatment of different diseases, along with specific applications and outcomes.

**TABLE 1:** Summary of various 3D printing techniques and their applications in the surgical treatment of spinal deformities and other complex spinal conditions.

References	Print technique	Patient population	Applications	Outcomes
Current study	Stereolithography (SLA)	38‐year‐old woman with complex spinal deformity, split cord malformation, hemivertebra, spina bifida, and tethered cord	Intraoperative navigation, selectin entry points and trajectories of screw	Reduced surgery time, reduced blood loss, Reduction of X‐ray dose
Wu et al. [9]	Not specified	50 patients with adolescent idiopathic scoliosis	Pedicle screw placement	Increased accuracy of screw placement and reduced radiation exposure
Tan et al. [13]	Fused deposition modeling (FDM)	23 patients with severe spinal deformities	Preoperative planning and intraoperative guidance	Improved pedicle screw placement accuracy and reduced operation time
Pan et al. [14]	Not specified	35 patients with spinal deformities	Pedicle screw placement and osteotomies	Reduced operation time and blood loss; improved surgeon confidence and osteotomy effectiveness
Ding et al. [15]	Not specified	8 adult patients with severe kyphoscoliosis due to TB, adolescent idiopathic scoliosis (AIS), ankylosing spondylitis (AS)	Safety enhancement during osteotomy	Increased safety during osteotomy procedures
Lador et al. [16]	Not specified	7 complex spinal oncology cases	Pre‐bent rod preparation and surgical simulation	Successful use of pre‐bent rods tested on models; improved surgical outcomes in oncology cases
Li et al. [17]	Stereolithography (SLA)	10 patients with complex spinal fractures	Surgical planning and implant design	Enhanced understanding of fracture patterns and improved surgical outcomes
Sugawara et al. [18]	Inkjet printing	20 patients with cervical spine tumors	Preoperative simulation and surgical planning	Improved surgical precision and reduced complications
Wang et al. [19]	Stereolithography (SLA)	4 patients with complex spinal disorders	Surgical planning	Shorter operating times, Reduced blood loss, Minimized risk of screw misplacement, Enhanced understanding of patient‐specific anatomy

### 5.1 Preoperative Planning

One of the major challenges in complex spine deformity surgery is understanding the three‐dimensional anatomy of the deformed spine. In some cases, the deformity is so intricate that it cannot be fully comprehended without a physical 3D model that displays the anatomical coordinates and spatial properties of the spine. 3D printing enables improved surgical planning by allowing neurosurgeons to engage with precise models of spine anatomy, aiding them in determining the best surgical options prior to the procedure.

### 5.2 Spinal Osteotomy

The use of 3D printing has been found to be superior to freehand techniques for pedicle screw placement in the spine and is also beneficial for performing osteotomies [13, 20, 21, 22]. Pan's study retrospectively compared two groups of 35 deformity patients (3D printed vs. freehand). In cases where the 3D model was used, operation time and blood loss were reduced, and the number of grade 3 to 6 osteotomies was higher in the mentioned group. Therefore, the study concluded that 3D printing increases the surgeon's confidence in performing better, more effective, and safer osteotomies [14]. In another study, 3D spine models were created for eight adult patients with severe kyphoscoliosis due to tuberculosis (TB), adolescent idiopathic scoliosis (AIS), and ankylosing spondylitis (AS), which ultimately increased the safety of osteotomy [15]. Other studies have used printed spine models for the correction of complex idiopathic scoliosis, congenital scoliosis, and hemivertebra resection, reporting positive results [23, 24, 25, 26].

### 5.3 Pedicle Screw Guide Plates

Several articles have reported the use of 3D printing to create guide plates that facilitate screw placement in patients with spinal deformities [25]. However, these guide plates require additional material for printing, which increases both time and cost. Nevertheless, the 3D spinal model itself can be used to simulate surgery and select the entry point and trajectory of the screws without the need for guide plates. We also avoided creating a guide plate for our patient and instead visually determined the entry points and the trajectory of the screws based on the 3D model.

### 5.4 Preparation of Pre‐Bent Rod

In a study by Lador, seven complex spinal oncological cases were 3D printed, with surgery simulated on five models. For two patients, the pre‐bent rod tested on the model was sterilized and implanted. As far as we have searched, this was the only study proposing this method [16].

### 5.5 Intraoperative Navigation

Most studies confirm the impact of 3D models in reducing time and blood loss during spinal deformity surgery [21, 27, 28]. Finding the exact anatomical location of the SCM during congenital scoliosis surgery has always been challenging. A significant amount of surgery time is spent locating the bony bar of the SCM and detethering the spinal cord. Many surgeons prefer to postpone deformity correction to a second stage. Therefore, a method that accurately identifies the location of the SCM can help reduce operation time and blood loss. It can even change a decision on two‐stage surgery into a single‐stage surgery. In our patient, the 3D model accurately showed the SCM's location within the vertebral canal and revealed the necessary geometry for accessing and resecting it through the dysraphic areas of the posterior elements.

### 5.6 Educational Utilization

The use of 3D‐printed models for training surgeons, residents, and medical students has gained increasing popularity in academic programs. Although dynamic and multi‐colored models enhance the quality of moulage, they significantly increase the production cost and time [29, 30, 31]. Therefore, producing such moulages is not justified for clinical purposes.

### 5.7 3D Printed Model Versus Neuronavigation

Neuronavigation is widely used in minimally invasive spine surgery and spinal deformity surgery, primarily for accurate pedicle screw placement. However, its popularity among surgeons has waned due to several limitations, including intra‐spinal space distortion during surgical positioning, poor quality of 3D fluoroscopy in obese or osteoporotic patients, and the high cost of equipment [32]. Despite these challenges, neuronavigation remains cost‐effective in centers with a high volume of complex deformity cases [33, 34]. While it enhances the accuracy and safety of screw placement, neuronavigation does not play a significant role in pre‐operative planning for deformity surgeries and osteotomies. Therefore, from this perspective, the 3D model is unique.

### 5.8 Reducing X‐Ray Dose in the Operating Room

According to the guidelines of the International Commission on Radiological Protection (ICRP) and the International Atomic Energy Agency (IAEA), medical groups must avoid unnecessary radiation exposure [35, 36]. One advantage of non‐X‐ray‐based navigation methods is the reduction of radiation dose to patients, doctors, and operating room staff. Studies have shown that 3D printing significantly reduces radiation dose during surgery [27]. In our experience, using a 3D model reduced the number of C‐arm shots to one‐third of those in usual deformity surgeries at our center.

### 5.9 Printing Technique

While a 3D‐printed spinal model unquestionably aids a surgeon in comprehending the patient's anatomy and in selecting the best surgical strategy, one of the primary drawbacks of physical models—compared to those on digital screens or augmented reality glasses—is model unity. In the context of 3D printing, model unity refers to the consistency and accuracy of the printed model in representing the actual anatomical structures. In our method, each pixel in the CT is classified as either bone or non‐bone based on expert user input, resulting in some osteosis regions not being classified or printed. However, multicolor 3D printing can significantly mitigate the shortcomings of model unity in understanding complex anatomical cases.

Various technologies exist for creating a 3D model, including stereolithography (SLA), inkjet printing, selective laser sintering (SLS), fused deposition modeling (FDM), and laminated object manufacturing (LOM) [12, 37, 38]. The production of a 3D model is time‐limited for emergency cases; preparing a computer‐aided design (CAD) file, printing, and post‐processing can take up to four days [38, 39, 40]. In our patient, these three steps took about 30 h.

For this patient, we used the SLA or resin 3D printing method, which utilizes a UV light source to harden liquid resin into plastic. The advantages of using this method in the medical field include smooth surfaces, high resolution, and accuracy.

It is highly recommended to print at a 1:1 scale. Although scaling down the model reduces production time, the amount of printer material, and thus the final cost, it may result in the loss of important anatomical details. Additionally, if screws are to be placed on the 3D model and a pre‐bent rod is to be created, maintaining the 1:1 scale is essential [16, 41].

### 5.10 Cost

Most articles emphasize the cost‐effectiveness of 3D printing for clinical and educational purposes [28, 30]. However, some studies highlight the high price of the 3D model as one of its limitations [42, 43]. To reduce the cost of 3D printing, we removed segments outside the curve of interest for surgery, utilized mono‐color resin material, and omitted printing additional guide plates. However, we do not recommend scaling down the 3D model for the reasons mentioned earlier.

## 6 Conclusion

Using 3D‐printed spine models in complex spinal deformity surgery offers significant benefits that enhance clinical practice and education. These models complement radiological assessments, enabling neurosurgeons to understand specific pathologies thoroughly beyond traditional 3D reconstructions.

## Author Contributions

**Hamed Hanif:** conceptualization, funding acquisition, supervision, writing – review and editing. **Reza Sharifi:** formal analysis, writing – original draft. **Negin Safari:** writing – original draft. **Nima Ostadrahimi:** writing – review and editing. **Alaaeldin Ahmad:** writing – review and editing.

## Consent

Written informed consent was obtained from the patient to publish this report in accordance with the journal's patient consent policy.

## Conflicts of Interest

The authors declare no conflicts of interest.

## Data Availability

The data that support the findings of this study are available from the corresponding author upon reasonable request.
